# The long-term outcomes and risk factors for precursor B-cell acute lymphoblastic leukemia without specific fusion genes in Chinese children: Experiences from multiple centers

**DOI:** 10.17305/bjbms.2021.5879

**Published:** 2021-08-10

**Authors:** Pinli Zou, Min Zhou, Jinquan Wen, Xin Liao, Yali Shen, Haiyan Liu, Lin Song, Jianwen Xiao

**Affiliations:** 1 Department of Hematology, Children’s Hospital of Chongqing Medical University, Chongqing, P.R. China; 2 Ministry of Education Key Laboratory of Child Development and Disorders, Chongqing, P.R. China; 3 National Clinical Research Center for Child Health and Disorders, Chongqing, P.R. China; 4 Department of Hematology, Chengdu Women’s and Children’s Central Hospital, Chengdu, P.R. China; 5 Department of Pediatric Hematology, Caihong Hospital of Xianyang, Shaanxi, P.R. China; 6 China International Science and Technology Cooperation base of Child Development and Critical Disorders, Chongqing, P.R. China; 7 Department of Pharmacy, Children’s Hospital of Chongqing Medical University, Chongqing, P.R. China; 8 Chongqing Key Laboratory of Pediatrics, Chongqing, P.R. China

**Keywords:** B-cell acute lymphoblastic leukemia, children, fusion genes, treatment response, minimal residual disease, next-generation sequencing

## Abstract

Specific fusion genes play important roles as risk factors in the pediatric B-cell acute lymphoblastic leukemia (B-ALL) population. In addition, these fusion genes are used for determination of patient treatment. However, the risk factors and long term outcomes in B-ALL patients without common fusion genes have not been well demonstrated, and thus our aim was to evaluate this patient population. We retrospectively analyzed clinical and laboratory findings, treatment responses, and outcomes in pediatric patients with B-ALL without specific fusion genes. Moreover, we analyzed whole-exome sequencing and/or RNA sequencing data from bone marrow (BM) relapsed patients. Overall, 283 patients were enrolled in the study. Traditional parameters and treatment responses at different time points (TP) were evaluated to classify risk groups and adjust treatment strategy. Statistical analysis showed that 49 (17.31%) patients relapsed, while treatment-related mortality was found in 11 (3.89%) patients. Ten-year prospective event-free survival (pEFS) was 78.2 ± 2.5%. Adverse and unreported genetic abnormalities were discovered in 25 BM relapse patients. Univariate analysis revealed that good responses of BM smears at TP1 and minimal residual disease (MRD) levels at TP2 and TP3 were strongly associated with prolonged pEFS. Moreover, multivariable analysis of outcomes and hazard ratios determined that positive MRD level was the key risk factor. The study results showed that traditional risk factors and poor prednisone response were overcome by modified chemotherapy. Next generation sequencing has proven to be a useful technique in identifying molecular risk factors in cases without common specific genetic alterations.

## INTRODUCTION

Acute lymphoblastic leukemia (ALL) is the most ­common childhood cancer, while precursor B-cell ALL (B-ALL) accounts for approximately 75-80% of newly diagnosed pediatric ALL cases [1]. B-ALL is a heterogeneous disease, and event-free survival (EFS) rates for this disease is more than 80% in developed countries with risk-adapted treatment strategies [1-3]. Specific chromosomal translocations and their fusion genes are detectable in approximately two-thirds of pediatric B-ALL patients, and such fusion genes play important roles as risk factors in strategic treatment. For example, B-ALL with *ETV6-RUNX1* and B-ALL with *MLL-AF4* were both classified as B-ALL with recurrent genetic abnormalities according to the WHO classification. However, the prognosis of B-ALL with *ETV6-RUNX1* is better than that of B-ALL with *MLL-AF4* [2,3]. Age at diagnosis, initial white blood cell (WBC) count, and abnormal chromosomal karyotypes remain the traditional risk factors for B-ALL patients without specific fusion genes, and studies have shown that early treatment response plays a key role in treatment strategy in these patients [2-4].

Fusion genes or mutated genes in ALL are traditionally detected by reverse-transcriptase polymerase chain reaction (RT-PCR). So far, more than 200 fusion genes or mutated genes have been detected in ALL patients [5,6]. However, it is difficult to detect all of the involved loci using RT-PCR. Next-generation sequencing (NGS) studies have provided valuable insights into the genomic alterations in malignancies using whole-exome sequencing (WES) and/or RNA sequencing (RNAseq), aiding clinicians in elaborating the rare genomic features of B-ALL [7,8].

In the present study, we assessed the outcomes and risk factors of pediatric B-ALL without specific fusion genes in three Chinese institutes. Subsequently, we performed WES and/or RNAseq in relapsed patients using the NGS technique to further evaluate risk gene mutations in pediatric B-ALL.

## MATERIALS AND METHODS

### Patients

We conducted a retrospective analysis that included 283 newly diagnosed pediatric B-ALL patients at Children’s Hospital of Chongqing Medical University (CHCMU), Chengdu Women’s and Children’s Central Hospital (CWCCH), and Caihong Hospital of Xianyang (CHX) between December 2009 and January 2015. The enrolled patients were <18 years at diagnosis. Exclusion criteria included patients with leukemia secondary to, for example, Down syndrome or myelodysplastic syndrome; Burkitt leukemia or mixed phenotype leukemia; patients with specific genetic abnormalities and/or fusion genes; and patients who had received chemotherapy before admission.

Patients from the cohort received bone marrow (BM) aspiration at initial diagnosis. The diagnosis of B-ALL was based on FAB classification and flow cytometry (FCM) [9]. Chromosomal karyotype was evaluated, and hyperdiploidy, hypodiploidy, complex karyotype, chromosome index, as well as other genetic abnormalities were defined according to the International System of Human Cytogenetic Nomenclature of 2009 (ISCN-2009) [10]. Between December 2009 and December 2012, *ETV6-RUNX1, TCF3-PBX1, BCR-ABL1*, and *KMT2A-AFF1* fusion genes were detected by split RT-PCR [11]. Fluorescence *in situ* hybridization (FISH) of *KMT2A* rearrangement (*KMT2Ar*) was also performed as reported in the literature [12]. Between January 2013 and January 2015, 43 common fusion genes were screened by multiplex nested RT-PCR, and the positive results were confirmed by split RT-PCR [13,14]. FISH of *KMT2Ar*, *MYC*, and *PDGFRb* was also performed [12,14]. The criteria for central nervous system leukemia (CNSL) or testicular leukemia (TL) were defined as described in the literature [2-4].

### Treatment, therapeutic evaluation, and risk strategy

The patients received chemotherapy according to the Chinese Children Leukemia Group ALL-2008 protocol (CCLG-ALL-2008, Table 1) [15,16], and all patients received intrathecal injection (iT) for the prophylaxis of CNSL as the protocol required; minimal residual disease (MRD) level monitoring was performed by FCM, and 10^−4^ was considered the limitation of MRD monitoring [15-17].

**TABLE 1:**
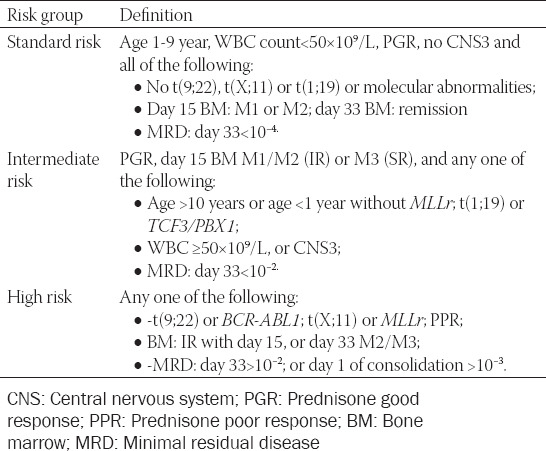
Stratification criteria by risk group

Peripheral leukemic cell counts were recorded after 7 days of prednisone induction, and prednisone good response (PGR) or prednisone poor response (PPR) was defined as peripheral leukemic cell counts <1 × 10^9^/L or ≥1 × 10^9^/L, respectively [15,16]. BM studies were conducted at 3 time points (TPs): Day 15 of induction remission (TP1), day 33 of induction remission (TP2), and before consolidation (TP3). At all three TPs, remission status of BM smear was recorded as follows: M1 (leukemic cells <5%), M2 (leukemic cells 5-25%), and M3 (leukemic cells ≥25%) [15,16]. The MRD level was monitored by FCM on TP2 and TP3 [15-17].

Traditional risk factors (age at diagnosis, WBC count, chromosome number, etc.) and treatment response were used to stratify patients into three groups [15,16]: A standard risk (SR) group, an intermediate risk (IR) group or a high risk (HR) group (Table 2). Details of these risk factors were listed at Supplemental material for this article and are available online.

**TABLE 2:**
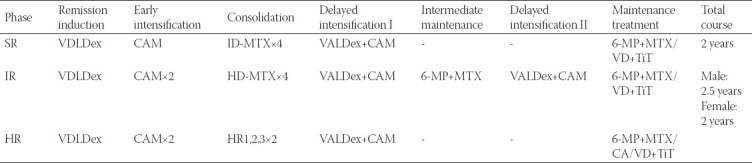
CCLG-2008 protocol for involved patients

### NGS for the relapsed patients

Tumor DNA samples of relapsed patients were obtained from BM at initial diagnosis and/or relapsed status, and germline samples were collected from the oral mucosa of patients and parental peripheral blood (PB). Genomic DNA was extracted using a QIAmp DNA Minikit (QIAGEN, China). Genomic DNA was enriched, and sequenced acquisition was carried out by exome probes (Agilent SureSelect Human All Exon V6); PCR amplifications of the whole exome were sequenced (Illumina HiSeq PE 150 bp). The original WES data were read using Illumina pipeline software (version 1.3.4) and searched in databases (dbSNP, 1000 Genomes Project, ClinVar, ESP6500, ExAc, Ensembl, HGMD, UCSC, etc.). Mutated genotypes were determined using GATK, LRT, MutationTaster, and SamTools software. Discovered variants were classified as described in the literature [18] through software analysis. Pathogenic genotypes and likely pathogenic genotypes were recorded as causal gene mutations, and causal gene mutations of tumor samples were confirmed by Sanger sequencing. Samples of the control group were cross-checked and detected by Sanger sequencing to identify somatic or germline causal gene mutations.

Total RNA was extracted from BM samples at relapse using a QIAamp RNA Blood Mini Kit (Qiagen, Cat.52304). An enriched and captured mRNA library was constructed and amplified by the KAPA mRNA HyperPrep Kit (KAPA/Roche, Cat. KK8581). PCR amplicons were sequenced via RNAseq using PE150 (Illumina HiSeq ×10), and the results were recorded.

### Ethical statement

The authors declare that there are no conflicts of interest. The study was approved by the Institutional Ethics Committee of CHCMU, and all data were fully anonymized (IRB No. 2019-151). An informed consent form was signed by the guardians of these patients.

### Statistical analysis

Complete remission (CR), BM relapse, or extramedullary relapse were defined as standard diagnostic criteria [15-17], while EFS was defined as the time from diagnosis to the date of last contact for EFS or to the first adverse event (relapse or refractory disease, death from any cause, secondary cancer, or loss to follow-up). Following December 2020, data about the clinical characteristics, laboratory findings, treatment-related mortality (TRM), and EFS of these patients were collected and retrospectively analyzed; NGS detection data of relapsed patients were also collected and analyzed. *SPSS 20.0* software (SPSS Inc., Chicago, IL) was used for statistical analysis, the impact of clinical and laboratory findings on EFS was assessed with the Kaplan-Meier method, and comparisons were made with the *log-rank* test. Multivariable analysis for prognosis and hazard ratio was performed using the Cox regression model. All probabilities reported were two-tailed, and *p* < 0.05 was regarded as significant differences.

## RESULTS

### Clinical characteristics

**FIGURE 1:**
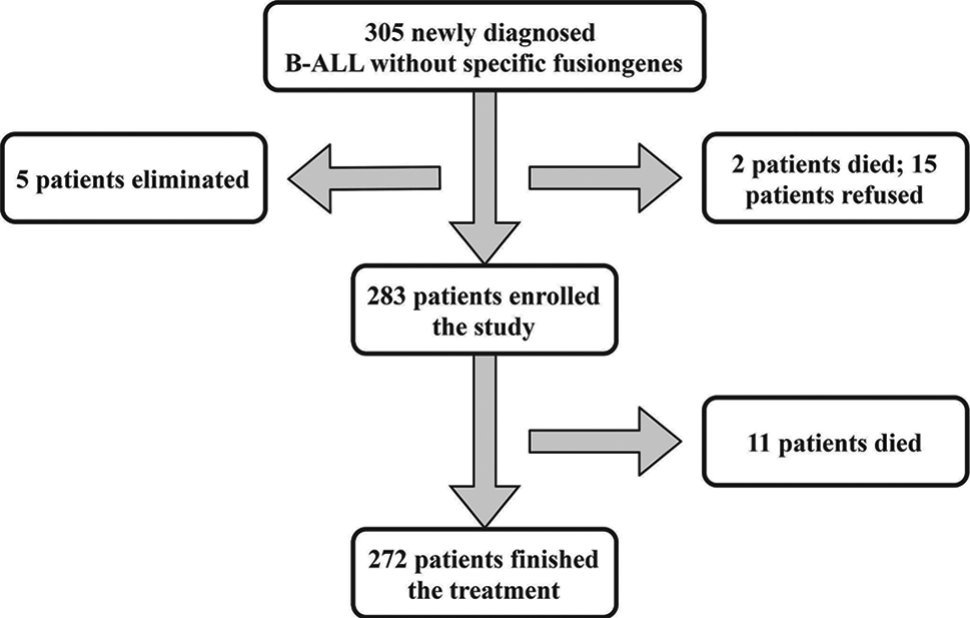
Patients enrolled in the study.

A total of 305 B-ALL patients without specific fusion genes were hospitalized in the study period, two patients died before diagnosis, 15 patients refused chemotherapy, five patients were eliminated due to errors in immunophenotyping at initial diagnosis, and 283 consecutive patients were treated with the CCLG-ALL-2008 protocol and were enrolled in the study (Figure 1).

The clinical and laboratory characteristics of the 283 enrolled patients, including 158 males and 125 females (1.26:1), are shown in Table 3. The average age at diagnosis and the initial WBC count was 4.91 ± 0.18 year and 17.74 ± 2.13×10^9^/L, respectively. Chromosomal karyotype was evaluated as follows: 147 (51.94%) patients presented with a normal karyotype (46, XX/46, XY), abnormal karyotypes were detected in 97 (34.28%) patients, and 39 (13.78%) patients were not analyzed. Hypodiploidy and pseudodiploidy/diploidy with translocation and hyperdiploidy were detected in 11 (11.34%), 13 (13.40%), and 72 (74.23%) patients, respectively; DNA index <1 was found in 10 (2.83%) patients. Chromosome number was ranged from 36 to 65, 4 patients with chromosome number <44, and 55 patients with chromosome number ≥50 were detected, while patients with near-haploid or triploid were not found in these patients.

**TABLE 3:**
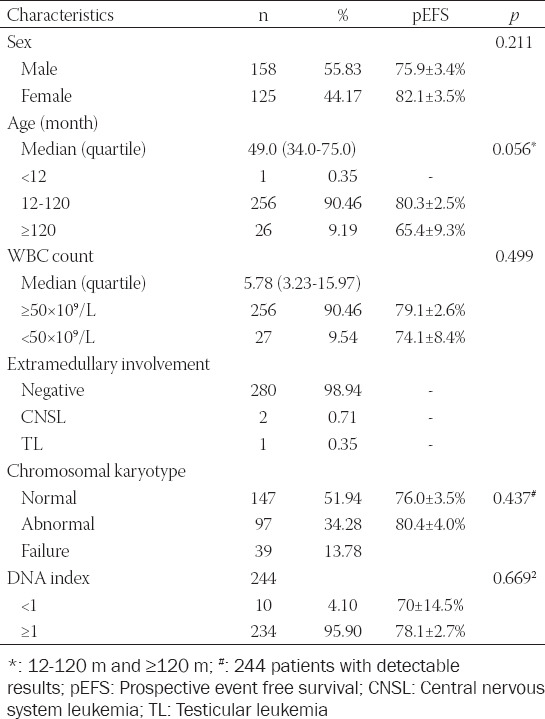
Characteristics of the 283 patients in the study

### Treatment response

Treatment response was evaluated as the protocol required, and risk groups were classified [15,16]. A total of 275 patients were defined as PGR, and eight patients were classified as PPR after 7 days of prednisone induction therapy. Remission status of BM smear was evaluated as M1, M2, or M3; at TP1, BM smears demonstrated 240 patients with M1, 36 patients with M2, and seven patients with M3. Two patients died before TP2, and BM samples were tested in 281 patients, showing that 273 patients were defined as M1, 8 patients were defined as M2, and the CR rate was 96.47% in the study group. The MRD level was also monitored at TP2; the results of 247 patients were negative and those of 34 patients (≥10^−2:^ 7 patients; ≥10^−4^-10^−2:^ 27 patients) were positive. Two patients died before TP3, BM samples were detected in 279 patients at TP3, and 278 patients and one patient were defined as M1 and M2, respectively. The MRD level was also monitored; 271 patients were negative and eight patients were positive (≥10^−2^: 1 patient; ≥10^−4^-10^−2^: 2 patients; ≥10^−4^: 4 patients), and the patient with M2 presented a negative MRD level (Table 4).

**TABLE 4:**
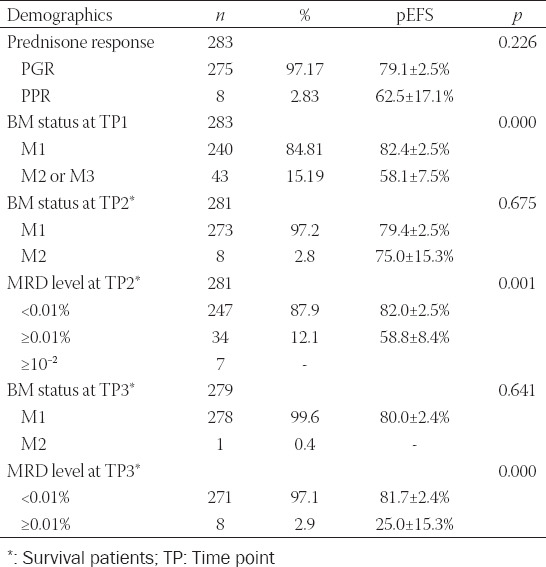
Treatment responses and outcomes

Classified by the risk strategy as required in the protocol [15,16], 279 measurable patients were divided into three risk groups (SR: 205 patients; IR: 66 patients; HR: 12 patients), and subsequent chemotherapy was continued; seven patients died of treatment complications, and 272 patients finished treatment.

### Prognosis and risk factors

**FIGURE 2:**
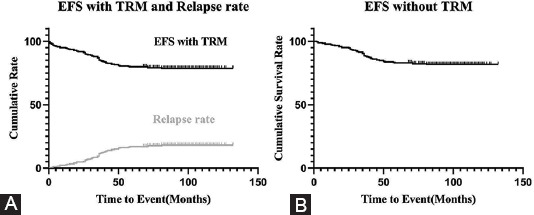
Survival of the cohort. (A) Total EFS rate and cumulative relapse rate of 283 patients: pEFS in the total of 283 patients was 78.2 ± 2.5%; the relapse rate was 17.31%. (B) EFS rate without TRM: pEFS in the 272 patients without TRM was 81.4 ± 2.4%; that of patients with TRM was 3.89%.

Following October 2020, 11 of 283 patients died of treatment complications, and the TRM rate was 3.89%; 49 patients relapsed, and the cumulative relapse rate was 17.31%. The 10-year prospective EFS (pEFS) rates for the cohort (283 patients) and 272 patients without TRM were 78.2 ± 2.5% and 81.4 ± 2.4%, respectively (Figure 2).

**FIGURE 3:**
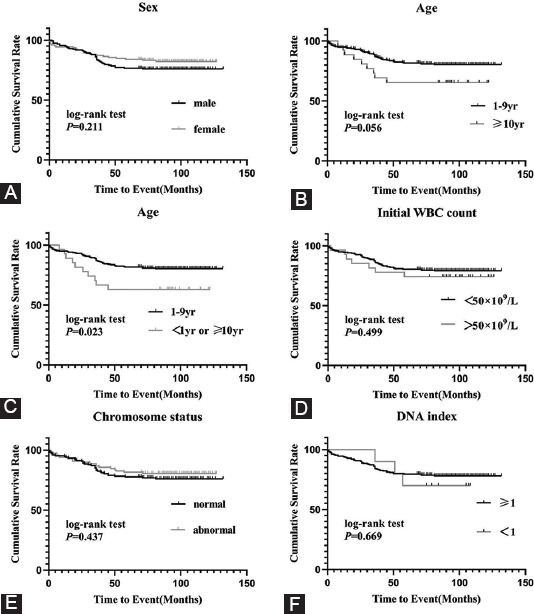
Relationships of traditional risk factors and prognosis. (A) pEFS rates between male patients and female patients groups: pEFS rates in males and females were 75.9 ± 3.4% and 82.1 ± 3.5% (*p* = 0.211). (B) pEFS rates between different age at diagnosis(excluding <1 year): pEFS rates in ≥10 year and 1-9 year groups were 65.4 ± 9.3% and 80.3 ± 2.5% (*p* = 0.056) (C) pEFS rates between different age at diagnosis(including <1 year): pEFS rates in <1 year + ≥10 year and 1-9 year groups were 63.0 ± 9.3% and 80.3 ± 2.5% (*p* = 0.023). (D) pEFS rates between different initial WBC count level: pEFS rates in WBC count <50 × 10^9^/L and ≥50 × 10^9^/L groups were 80.3 ± 2.5% and 65.4 ± 9.3% (*p* = 0.499). E: pEFS rates between different chromosome status: pEFS rates in normal and abnormal chromosomal karyotype groups were 76.0 ± 3.5% and 80.4 ± 4.0% (*p* = 0.437). F: pEFS rates between different DNA index: pEFS rates in DNA index ≥1 and <1 groups were 78.1 ± 2.7% and 70 ± 14.5% (*p* = 0.669).

The relationship of traditional risk factors and the prognoses of the study subjects are summarized in Table 3 and Figure 3. Statistical differences were not found in the EFS of traditional risk factors, including sex (*p* = 0.211), initial WBC count (*p* = 0.499), chromosome status (*p* = 0.437), or DNA index (*p* = 0.669). There was no difference of pEFS in these cases 1-10 year compared with these ≥10 year (*p* = 0.669). There was no differences of pEFS in these cases 1-10 year compared with these ≥10 year (*p* = 0.056), but Figure 2 also shows that the pEFS of children <1 year or ≥10 year is lower than that of children between 1 and 9 years (*p* = 0.023); only one patient <1 year was enrolled in the study. Four patients with near-haploid or low-hypodiploid karyotypes were enrolled in the study, and two of them relapsed. However, the data were limited to summarize the relationships of the outcomes.

**FIGURE 4:**
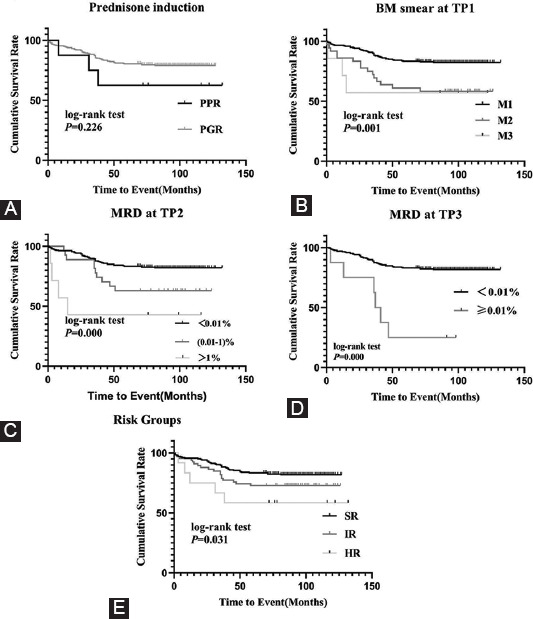
Outcome relationships of treatment response and risk groups. (A) pEFS rates between PGR and PPR patient groups: pEFS rates in PGR and PPR patient groups were 79.1 ± 2.5% and 62.5 ± 17.1% (*p* = 0.226). (B) pEFS rates between M1, M2 and M3 groups at TP1: pEFS rates in M1, M2 and M3 groups TP1 were 82.4 ± 2.5%, 60.0 ± 8.3% and 57.1 ± 18.7% (*p* = 0.001). (C) pEFS rates between different MRD level at TP2: pEFS rates in MRD <0.01 × 10^−2^, 0.01-1 × 10^−2^ and ≥1 × 10^−2^ groups at TP2 were 82.0 ± 2.5%, 63.0 ± 9.3% and 42.9 ± 18.7% (*p* < 0.001). (D) pEFS rates between different MRD level at TP3: pEFS rates in MRD <0.01 × 10^−4^ and ≥0.01 × 10^−4^ groups at TP3 were 81.7 ± 2.4% and 25.0 ± 15.3% (*p* < 0.001). (E) pEFS rates between different risk groups: pEFS rates in SR, IR and HR groups were 81.8 ± 2.7%, 72.7 ± 5.5% and 8.3 ± 14.2% (*p* = 0.031).

The outcomes and treatment responses of the risk groups are shown in Figure 4 and Table 4. Statistical differences were not found in the EFS of PGR and PPR patients (*p* = 0.226), but only eight patients with PPR were involved in the study. The patients with M1 at TP1 were strongly associated with prolonged mean survival time in comparison to the patients with M2 or M3 (*p* = 0.001). Statistical differences were also found for MRD monitoring at TP2 (*p* < 0.001) and TP3 (*p* < 0.001). Compared with the IR or HR group, patients in the SR group presented with longer survival times and lower relapse rates (*p* = 0.031).

Multivariable analysis for prognosis and hazard ratio was performed using the *Cox* regression model (Table 5), and it revealed that traditional risk factors or PPR were removed by the modified chemotherapy strategy; however, larger samples and multicenter studies are needed to confirm this hypothesis. It seems that the MRD level was the key risk factor in pediatric B-ALL patients without specific fusion genes.

**TABLE 5:**
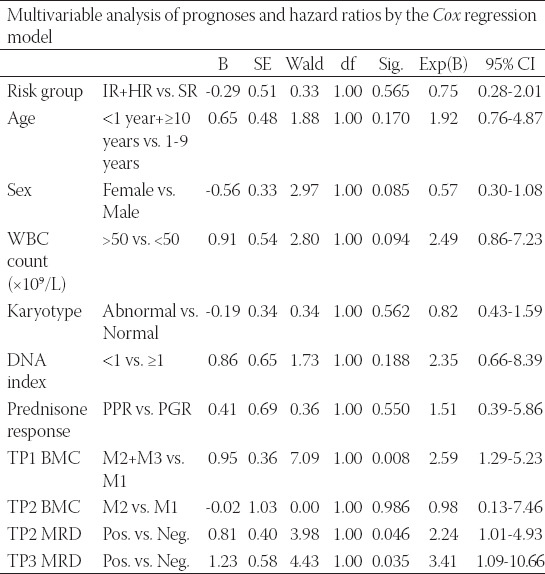
Multivariable analysis of prognoses and hazard ratios by the *Cox* regression model

Mortality occurred in 11 of 283 patients, including ten with sepsis and one with Pneumocystis carinii pneumonia (PCP). TRM occurred in four patients with remission induction, five patients with early intensification or consolidation, and two patients on maintenance treatment.

### NGS for relapsed patients

A total of 49 patients relapsed (39 with BM relapse; 7 with CNS relapse; 1 with testicular relapse; and 1 with both CNSL and BM relapse). BM samples were acquired from relapsed BM patients, and NGS was performed. WES sequencing was performed for 25 patients at diagnosis and/or on relapse, and RNA-seq was performed for 6 patients at relapse; the data are presented in the Supplementary Materials. Germline mutations were detectable by WES sequencing in 2 patients (*TP53* or *FLT3*); somatic mutations were confirmed in 14 or 15 patients at diagnosis or relapse; and adverse mutations, including *TP53*, *CREBBP* and *IKZF1*, were identified in five patients. RNAseq results were evaluated, and molecular abnormalities were found in six patients; reversed molecular abnormalities were found in three patients (*CREBBP*, *TP53*, and *P2RY-CRLF2*), and unreported genetic abnormalities in B-ALL, such as *TPM4-KLF2* or *NR3C1-CDC42* transcript, were also detected (available in the Supplementary material). NGS data for these BM relapsed cases were listed in the Supplementary material.

## DISCUSSION

B-ALL is the most common subtype of pediatric ALL. Contemporary protocols using risk-adapted treatment strategies have effectively improved the prognosis of pediatric B-ALL [2,4,6]. The ten-year pEFS in the study reached 80%, similar to the rates reported in developed countries [6,11,17]. TRM in the study was nearly 4%, which indicated that infection remained an important adverse event in ALL populations in developing countries. Part of the possible causal factors are poor economic conditions and giving-up treatment in developing countries [15].

Traditional risk factors, including sex, age, WBC count, and DNA index, were assessed in the study, but significant differences were not found in the pEFS for these risk factors except age (data were limited). This revealed that these traditional risk factors could be eliminated by modified intensive chemotherapy [2,4,6]. Fifty percent of patients with near-haploid or low-hypodiploid karyotypes in the study relapsed, similar to the literature reports [19,20]. Patients with hyperdiploidy were detected in 72 of 97 (74.23%) patients with abnormal karyotypes, we think it attributed to the fact that patients with specific fusion genes were excluded in the study, and chromosomal karyotype was failure to analyze in a few patients in the study. However, the data were limited to the number of cases, and large-sample and multicenter studies are needed to confirm the inference.

Many studies have shown that early treatment response is a strong outcome indicator for pediatric ALL [21-24]. EFS was higher in PGR patients than in PPR patients [15,16,21,22], and eight patients with PPR appeared in the study. However, a similar result was not observed in patients who underwent intensive treatment, which revealed that risk factors might be overcome by intensive treatment, though further research is still needed. MRD levels have been highlighted as an important predictor of survival in pediatric ALL populations, and patients with early and continuous negative MRD levels present with a satisfactory outcome [15,17,23]. In this study, a positive MRD level at TP2 or TP3 was related to a poor outcome, which indicated that the MRD level is an important predictor of survival in ALL patients without specific fusion genes.

With the development of molecular cytogenetics in malignancies, an increasing number of specific chromosomal translocations and their fusion genes have been discovered in B-ALL patients. More than 200 specific fusion genes have been discovered in pediatric B-ALL populations, and such genes are playing an increasingly important role in strategic risk treatment and serve as an index of MRD monitoring [3,5,25]. *ETV6/RUNX1, TCF3/PBX1, KMT2Ar*, and *BCR/ABL1* fusion genes were frequently detectable in B-ALL patients [8,11], and B-ALL patients with *ETV6/RUNX1* experienced favorable prognosis, whereas those patients with *KMT2Ar* endured poor results. Patients with *TCF3/PBX1* who received intensive chemotherapy had an improved prognosis [15,16]. Patients with *BCR-ABL1* continued to experience a poor outcome, but survival improved with combination treatment with chemotherapy and tyrosine kinase inhibitor [14,26].

Specific fusion genes have yet to be clarified in at least one-third of B-ALL patients [5,7,8], and our study and the literature verified that early treatment response was the main prognostic factor in these patients [4-6,17,21-24]. In recent studies, NGS has been used to discover more genetic abnormalities in B-ALL, and advanced studies are exploring the relationships among these genetic abnormalities, pathogenesis, and outcomes [25,27-29]. In this study, NGS detected abnormalities in 62.5% of BM relapsed patients, rare or unreported fusion genes and/or gene mutations were detectable, and adverse molecular genetic abnormalities such as *TP53, CREBBP*, and *IKZF1* [30,31] were detected in BM relapsed patients. These findings suggest that the application of NGS will reveal more molecular genetic changes in B-ALL patients without specific fusion genes than the use of traditional detection means, meaning the outcomes of these patients could be further improved by a modified strategy [25,32]; NGS and other technologies were also identified that genetic mutations were different at initial and relapsed B-ALL samples [31], it revealed the evolution from diagnosis to relapse and treatment should be adjusted according to it. However, due to limited RNA-seq data of relapsed cases, common adverse fusion genes including Ph-like were not detected. The detection of molecular genetic abnormalities will serve to monitor the MRD [33,34], and the targeting of signaling pathways will be further researched [35,36].

## CONCLUSION

In the present study, we assessed the outcomes and risk factors for Chinese pediatric B-ALL patients without specific fusion genes. The assessment demonstrated favorable outcomes in the cohort. The study results show that traditional risk factors were overcome by modified chemotherapy and that the positive MRD level was a key risk factor in pediatric B-ALL. NGS has proven to be a useful technique to identify other molecular genetic risk factors in cases without common specific genetic alterations.

## Figures and Tables

**TABLE 1:**
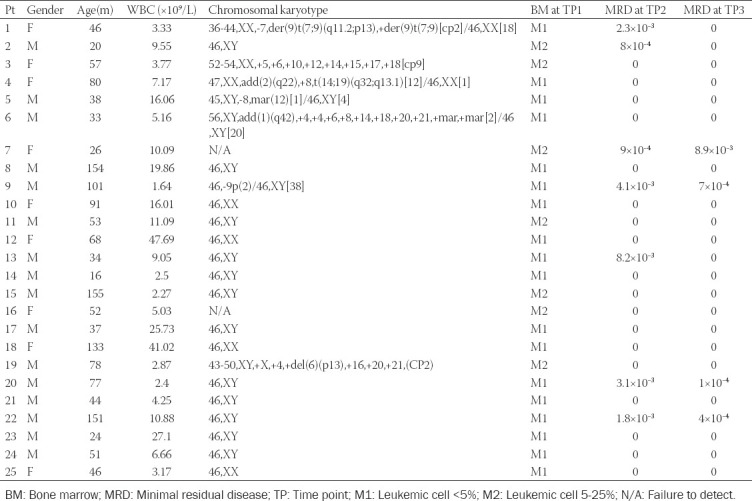
Character of 25 bone marrow relapsed patients

**TABLE 2:**
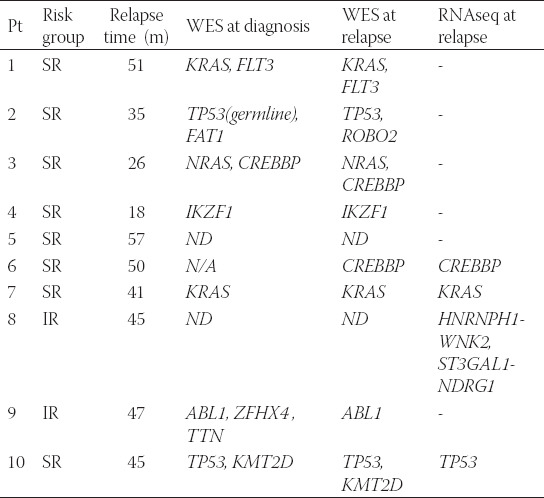
Next generation sequencing results of bone marrow relapsed patients
